# Training and Competition Load in Female Basketball: A Systematic Review

**DOI:** 10.3390/ijerph17082639

**Published:** 2020-04-12

**Authors:** María Reina, Javier García-Rubio, Sergio J. Ibáñez

**Affiliations:** Training Optimization and Sports Performance Research Group (GOERD), Sport Science Faculty, University of Extremadura, 10005 Caceres, Spain; jagaru@unex.es (J.G.-R.); sibanez@unex.es (S.J.I.)

**Keywords:** performance analysis, load, women, basketball

## Abstract

The scientific literature on women’s basketball is still limited, mainly in performance parameters. The purpose of this study was to analyse the state of art on the internal and external loads supported by female basketball players during sports practice. The design of this research is theoretical. The most relevant databases were searched for pertinent published studies according to the following keywords: “basketball”, “female” or “woman”, “training” or “competition”, “load” or “demand”. Of the 644 studies initially identified, 26 were selected for a complete review. These investigations were characterised by having as an objective an individualization of training for this type of population. Of the selected studies, it was evaluated: (i) goal (training, competition or both); (ii) category (stages: U14, U16, U18 and senior; level: state, national or international); (iii) type of load (Internal, External or both); (iv) instruments used and (v) variables analysed. The most studied goal was competition, mainly in senior national level, carrying out an analysis of the external and internal load together. Depending on the instruments and the variables used, the subjective load analysis was recurrent and important in the publication of articles in this topic. The quality of the studies was good, but for a better description of women’s basketball, there is a need to jointly investigate sports training and competition, to study the training categories and to use micro technology that guarantees obtaining objective and reliable data.

## 1. Introduction

Team sports, such as basketball, are difficult to analyse due to their unpredictable and intermittent nature [1]. Basketball is composed of technical-tactical skills, which have a direct influence on the requirements supported by players during the competition [2,3]. Therefore, the final performance in the game depends directly on a large number of variables with different orientations. There are many studies that consider that basketball has a hybrid character, in which the greatest amount of mobilized energy comes from the aerobic route [4,5,6]. However, explosive actions, such as changes of direction, jumps, or movements at maximum intensity, depend on the anaerobic routes. These kinds of actions are determinant for the athlete’s final performance [5,6,7,8].

**Figure 1:**
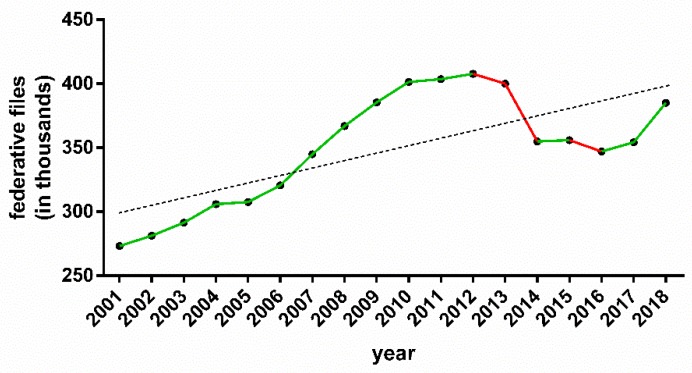
Evolution of the number of basketball players in Spain in recent years.

Currently, given the number of participants, basketball can be considered one of the most popular sports in the world. The increase in the number of high-level female athletes, amateurs and those in training stages has been gradually consolidated in recent years in Spain (Figure 1), in addition to the support of national and international institutions for women’s sports. In the field of research, despite having experienced progressive growth in the last 10 years, a volume of scientific production as consolidated, as in the case of male basketball or male sports in general, has not been reached.

Thus, the scientific community is beginning to show a special interest in studies that regard factors of performance and load involved in this sport. Load control in basketball requires scientific information to establish analytical assessment protocols. The most common demand analysis in basketball has been (i) analysis of internal load through heart rate and subjective scales; and (ii) external load analysis by time motion analysis [9,10]. Previous research has already reported on different models for load analysis in training and matches, mainly from an internal analysis (physiological variables) and through Time Motion Analysis (video analysis), mostly in men’s basketball [4,5,11,12].

As for women’s basketball, the first women’s basketball game was played in 1893, when Senda Berenson adapted James Naismith’s basketball rules for women. The professional Women’s National Basketball Association (WNBA) was founded in 1996, attracting a great deal of interest worldwide. Conversely, the scientific literature on women’s basketball is still limited, particularly regarding performance parameters (3, 8, 21). The most researched object of study in women basketball players is injury. In addition to this, the female gender has been studied through hormonal, biological and anatomical factors, defining the characteristics of the athletes, but not how to work with them. Boles and Ferguson [13] present female athletes as a unique challenge for sports medicine, since they are at a higher risk than male athletes of suffering some injuries, which are related to their morphological and physiological differences. To solve this, it is important to investigate training and competition in this type of population as their physical conditioning has been characterised from male data (5). Therefore, optimizing performance in women’s basketball is necessary to respect sports training principles, such as individuality and specificity [11,14].

Therefore, although there are large numbers of high-level women athletes, amateurs or players in the training stages, no research is specifically focused on their characteristics. That is why better description of women’s basketball is, thus, warranted. There are different methods to facilitate the description and analysis of the athletes’ load during training and competition. Consequently, an updated systematic analysis of the literature is necessary for the following reasons: (i) the previous reviews [3,15] did not analyse activity demands relative to training sessions or categories. (ii) There are no review articles that study only women’s basketball; this is necessary to be able to work on the specificity of training and competition in this modality. With an increase in the number of available studies, separate analyses considering these factors can be made that provide important practical applications for implementation by basketball coaches, athletic trainers, strength and conditioning practitioners, and sports scientists in women’s basketball. (iii) New, important indicators of the load imposed on players, such as the variables derived from inertial devices, have only been measured in recent studies and, thus, warrant consideration in a systematic analysis. (iv) Given the increased research attention to women’s basketball in recent years, an updated review is needed to include studies of female players. (v) Our review encompasses more recent research reports of the physical and physiological responses experienced during basketball match play and training sessions, potentially allowing for a more precise development of optimal training approaches to achieve desired performance in current players.

To the best our knowledge, no previous research has systematically verified the literature to identify the demands of female basketball players during training and competition. Men’s basketball is the most investigated; thus, there is an important gap in the available research that does not allow us to conclude whether women’s basketball has the same needs. For example, in the last review published, one of the exclusion criteria was that the sample be women [16]. In this context, the purpose of the present study was to provide a systematic review to analyse the state of art on the loads supported by female basketball players, according to competition level, type of load, training or competition, variables analysed and instruments used.

## 2. Materials and Methods

### 2.1. Study Design and Search Strategy

#### 2.1.1. Study Design

The study is a systematic literature review. The design of the research is theoretical [17], compiling those investigations whose subject of study is the load supported by female basketball players during training and competition. Data were collected through a systematic development of the methodology for the selection of articles, definition of variables, coding and data analysis, etc. The PRISMA methodology was used for the development of the research (Preferred Reporting Items for Systematic reviews and Meta-Analyses) [18]. The research characteristics that follow from this methodological proposal are: definition of the objectives, explicit and reproducible methodology; systematic search for evidence following the eligibility criteria; assessment of the validity of the findings; and systematic presentation and synthesis of the characteristics and findings of the included studies.

#### 2.1.2. Search Strategy

The Web of Science electronic database (WOS), Scopus and PubMed were searched on 11 November, 2019, using keywords. For its search, the following terms and Booleans were used: Basketball AND (Female OR Woman) AND (Training OR Competition) AND (Load OR Demand). The key words were used next to the logical operator “AND” and “OR”. The initial search was conducted by a researcher and 644 articles were obtained. Then, the duplicates were eliminated and an intensive review of all the titles and abstracts obtained was carried out. For the final selection process, the full version of the remaining articles was read and studies not related to the subject of review were excluded. Finally, a qualitative analysis of the studies included in the review was performed.

### 2.2. Inclusion and Exclusion Criteria

This review included original articles that considered female basketball players. The study participants were classified into four groups: U15, U16, U18 and senior. In addition, they were classified in three competitive levels: state, national and international. In the analysis of load demands, a distinction was made between training and competition, or both. The type of load analysed (external, internal or both) and the type of measures and variables analysed were differentiated. This demand analysis had to be completely ecological, excluding those studies where tests or field tests, training programs, etc., were performed.

Therefore, the studies included in this review met the following criteria: (i) the study was published in English or Spanish; (ii) the study included internal, external or both load variables during training and/or competition in basketball; and (iii) the participants were women of all categories and levels. Studies were excluded if (i) the study participants were men; (ii) the study participants were wheelchair basketball players; (iii) The participants were referees; (iv) the data collected did not describe the training or competition demands; and (v) the study consisted of a review or field tests.

### 2.3. Quality of the Studies

To evaluate the quality of the studies, quality criteria were used to analyse the quantitative publications [19] and composed of 16 items in an evaluation process performed by five senior researchers with an academic range of PhDs in sports science, and a high number of valuable publications on this topic. Articles were assessed based on purpose (Q1), background (Q2), design (Q3), sample (Q4 and Q5), informed consent procedure (Q6), outcome measures Q7 and Q8), method description (Q9), significance of results (Q10), analysis (Q11), practical importance (Q12), dropouts (Q13), conclusions (Q14), practical implications (Q15) and limitations (Q16). All 16 quality criteria were scored on a binary scale (0/1), wherein two of those criteria (Q6 and Q13) presented the option: ‘If not applicable’. To make a fair comparison between studies of different designs, the decision was taken to calculate a percentage score as a final measure of methodological quality [20]. All articles were classified as (i) low methodological quality, with a score ≤50%; (ii) good methodological quality, with a score of between 51% and 75%, and (iii) excellent methodological quality, with a score >75%.

### 2.4. Data Extraction

The protocol carried out for the definition of the characteristics of the articles included the following: year, goal, category, level, type of load, instruments and variables analysed.

Year: date of article publication.

Goal (G): three types of objectives were established: (i) competition analysis (C). The load supported by the players during official matches; (ii) training analysis (T). The load supported by the players during the tasks carried out in the training sessions, without a goal of specific improvement or modifications for the achievement of the same. (iii) Analysis, both competition and training (B).

Category (C): the women’s basketball studies were classified in different formative stages: (i) U15 (14–15 years old); (ii) U16 (15–16 years old); (iii) U18 (17–18 years old) and senior (>18 years old).

Level (L): the competitive level is classified in: (i) State (S): competing in regional leagues; (ii) national (N): high level competing nationally or (iii) international (I): high level competing internationally.

Type of Load (TL): the work load that the athletes support, either in training or during the competition is, according to González-Badillo and Serna [21], “The set of psychological and biological demands (internal or real load) caused by the training activities or competition (external load or proposal)”. According to Schelling [9], the load is divided into: (i) external load (EL): the load supported by the athletes depends on volume (time and distance), intensity of actions, duration and density of efforts and pauses, analysis of the actions (jumps, steps, impacts, sprints, accelerations, etc.) and volume of the muscles involved. The external load values obtained can be significantly influenced by gender, player level, the game position, the moment of the game and the style of play. (ii) Internal load (IL): the load supported by the athletes depends on heart rate, maximum oxygen consumption, lactate concentration, enzymatic parameters, modification of minerals and ions, hormonal alterations or other biochemical variations. Internal load values vary according to gender, age, player level, playing position, match time, play style, emotional state and circadian rhythm, as well as other modulating factors such as race, body composition, meteorology, nutrition, sleep and analysis technique.

Instruments (I): the following instruments were used by the researchers to collect the data; (i) internal load measurements: heart rate bands (HRB), blood markers (BM), subjective scales (SS), etc.; (ii) external load measurements: time motion analysis (TMA) and microtechnology-like inertial devices (ID), Global Positioning System (GPS) devices, accelerometers (AC), etc.

Variables (V): the variables analysed in the computation of studies [10,15] and which all the research in this field is using today are as follows:

(i) Internal load variables: (a) heart rate (HR): measured in beats per minute. The values have been expressed as average heart rate (HRavg), maximum heart rate (HRmax), percentage (%) maximum heart rate (% HRmax) and work zones. (b) Blood lactate concentration (LAC): obtained from blood samples taken from athletes by subcutaneous puncture and analysed automatically in a portable instrument that interprets lactate values. (c) Subjective load (SL): scales, interview, fatigue, questionnaires, etc.
(ii) External load variables: (a) distance (D): meters or steps travelled by the athletes throughout the activity analysed. (b) Jumps (J): movement that consists of elevating oneself from the court with an impulse that implies more than 400 ms of flight, in order to land in the same place or another. The time from the start of the jumping action to the completion of landing; any movement or activity whereby a player initiates a jumping action and feet break contact with the playing surface; players spring into the air using a one- or two-leg take-off. (c) Speed profile (SP): forward movement at a high intensity characterised by effort and purpose at close to maximum, multidirectional movement performed at velocities of >7 ms^−1^. (d) Accelerometry (ACC): acceleration data, interpreted as external load, are obtained from a tri-axial accelerometer. The straight addition of the instantaneous change of rates of resultant accelerations (also known as jerk) over time represent the acceleration load for a drill or activity. (e) PlayerLoad™: accumulative triaxial (anteroposterior, mediolateral and vertical) g-force alterations produced by an athlete. (f) Subjective load (SL): the training load is evaluated from surveys or subjective scales of effort. (g) Movement (MOV): extracted match activity outcome measures were defined and classified into the following categories: standing/walking, jogging or low speed running, running or moderate speed running, striding or high-speed running and sprinting or maximal speed running; technical actions, such as changing direction, dribbling, upper body movements, passes and static exertion. Finally, rest has also been analysed.

### 2.5. Statistical Analysis

A descriptive analysis was made of all the variables recorded in the research (frequency and percentage). A multiple response analysis was carried out on the following variables: goal, level, load, instruments and variables. These variables were coded as different if more than one record was identified. The analysis was completed with contingency tables and figures to identify the relationships between the research variables [22]. Regarding the qualitative analysis of the studies, the degree of agreement among observers was assessed for 100% of the studies (*n* = 26) given by the value of Cohen’s kappa [23].

## 3. Results

### 3.1. Study Selections

**Figure 2:**
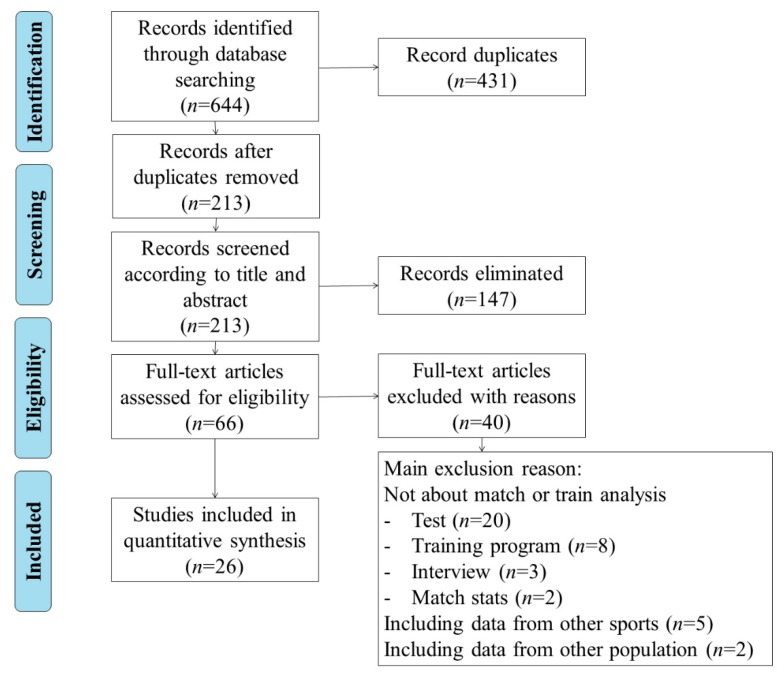
Flow chart.

Figure 2 shows the article selection process. The articles were included as long as they met a series of inclusion requirements. Four phases were followed: (i) identification; (ii) screening; (iii) eligibility; and (iv) inclusion. The initial search identified 644 titles in the described databases: WOS, Scopus and PubMed. These data were then exported to reference manager software (EndNote X8), and any duplicates were eliminated automatically (431 references). The remaining 213 articles were then screened according to the title and abstract for relevance, resulting in another 147 studies being eliminated from the database. The full text of the remaining 66 articles was read and another 40 were rejected due to a lack of relevance for the specific purpose of the current study. At the end of the screening procedure, 26 articles received further in-depth reading and analysis for the systematic review (attached Table S1).

The main reason for exclusion was that a published study did not concern match or training analysis (*n* = 33). Other reasons for exclusion were (i) data were included from other types of formats, such as reviews, conferences, abstracts, etc., (ii) from other types of team sports and (iii) from other populations (male gender or referees) (*n* = 7).

**Figure 3:**
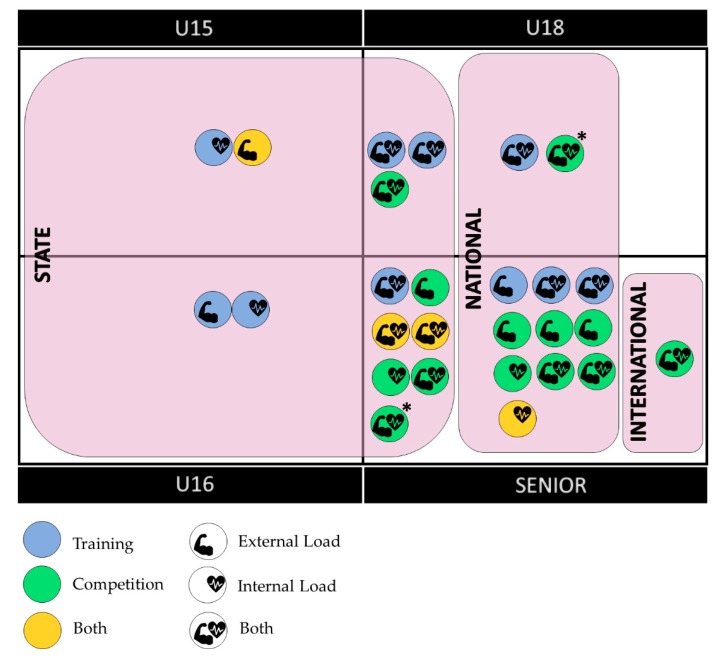
Grouping of the articles reviewed. Category (u15, u16, u18, senior); Level (state, national, international); Goal (Training, competition, both); Load (Internal, external, both). * In the same, there are u18 and senior samples.

Twenty-six scientific articles were identified for this systematic review whose main focus of study was the analysis of the load in women’s basketball. Figure 3 shows main variables in a visual way, depending on the category, level, goal and load.

Depending on the category, most of the studies included senior players (*n* = 18). In terms of level, the national category is the most studied (*n* = 14). Increasingly, research implements analyses of both internal and external loads together (*n* = 15), especially in competition (*n* = 12).

### 3.2. Results by Year

**Figure 4:**
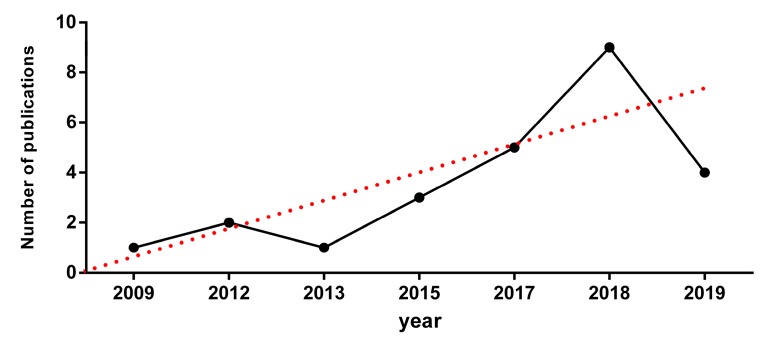
Publications in the last few years.

**Figure 5:**
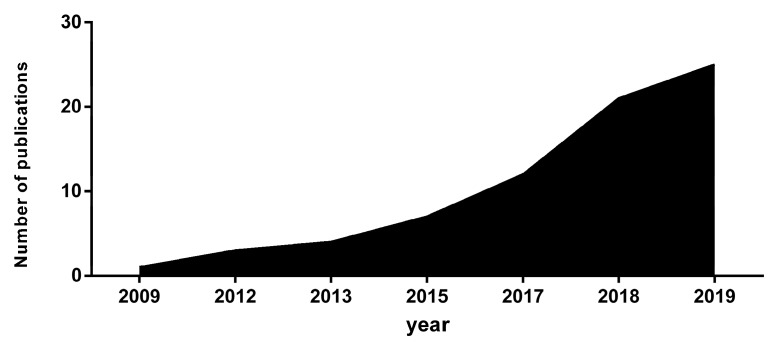
Accumulated publications.

In recent years, there has been an increase in the publication of articles on this research topic. The first article was published in 2009, since then, the article publication has increased. In 2018, eight articles were published. There is a linear progression in the production of these articles, increasing (r^2^ = 0.7476), in the period analysed (Figure 4 and Figure 5).

### 3.3. Results by Goal, Level and Type of Load

**Figure 6:**
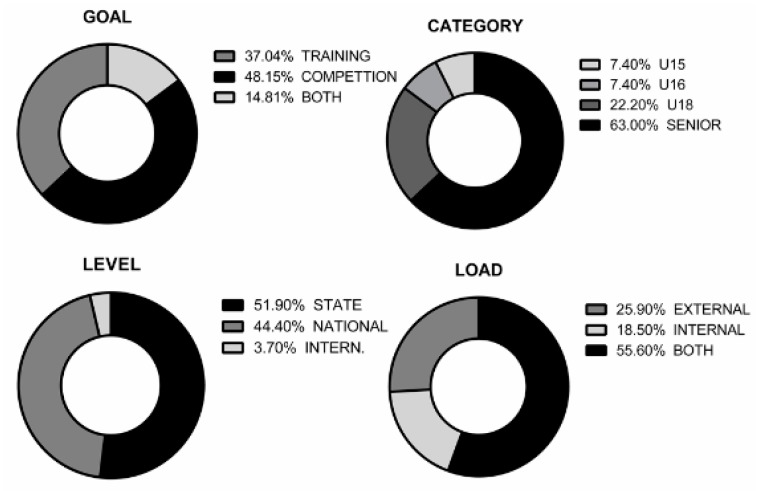
Distribution of the main variables.

Figure 6 shows that, the most studied object was competition; 42.31% of the studies reviewed analyse the competition, 38.46% the training and 19.23% both. Regarding the category analysed, it is found that most of the studies carried out are with a senior sample (66.7%), while in training categories there is not enough evidence yet. Depending on the level of competition the studies are at, state (44.4%) and national (51.9%) levels, while at the international level, only one study was found. The type of load analysed in these studies as 20% internal load and another 20% external load, independently. Moreover, both were analysed in 60% of cases. The most studied objective was competition (40.7%), mainly in national categories, carrying out an analysis of the external and internal load together (55.6%).

Table 1 shows the relationships identified between three main classification criteria of the study, in order to relate to each other: Goal, Level and Load Type.

As a main result, depending on the goal, it is observed that the training is more commonly analysed in-state levels (60%), while at national levels they focus on the study of the competition. Regarding the type of load, 50% of studies analysed internal and external load during training, while 54.5% of studies analysed both loads during competition. The most relevant outcomes were found in the studies that investigated, jointly, both contexts (training and competition) and both types of load (internal and external) (66.7%).

### 3.4. Results by Variables and Instruments

**Figure 7:**
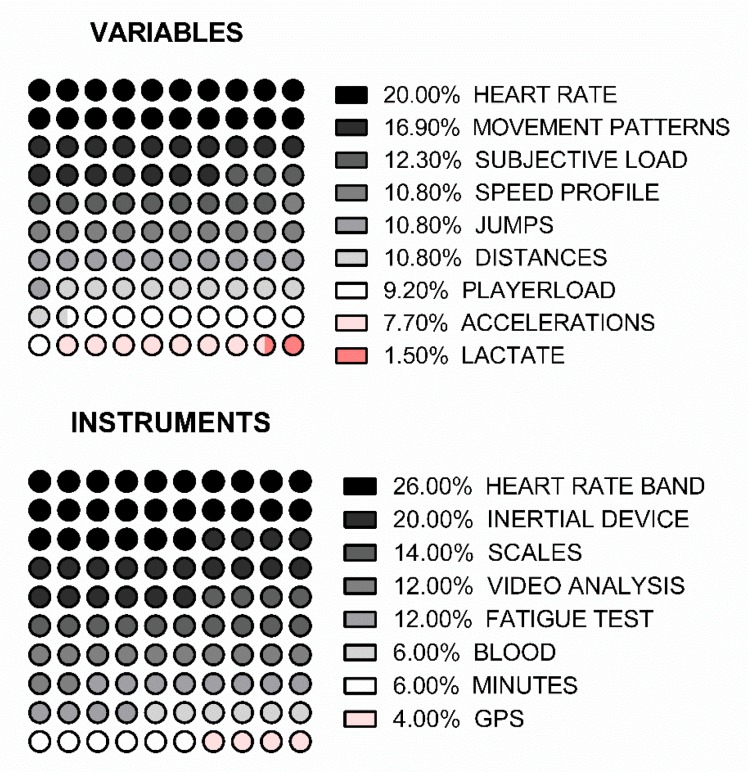
Distribution of instruments and variables.

The most commonly used variables in the reviewed articles are heart rate and movement patterns (21.2% and 16.7%, respectively). Therefore, the instruments most used by the authors are heart rate bands (devices that allow you to measure heart rate using a chest band) and subjective scales (28.6% and 30.6%, respectively) (Figure 7). The subjective load analysis is recurrent and important in the publication of articles in the sports field due to its low cost and the immediacy in the evaluation of the results.

Table 2 and Table 3 show the relationships identified between the variables analysed and instruments used by Goal of study.

The main variables analysed during training were the heart rate (60%) from heart rate bands (60%) and subjective loads (50%) from scales and questionnaires (70%). However, during the competition, the analysis of movement patterns (63.6%) takes more presence from video analysis (36.4%).

### 3.5. Quality of the Studies

To analyse the quality of the selected studies, the classification designed by Law, Stewart, Pollock, Letts, Bosch and Westmorland [17] was utilised. The result of the inter-coders reliability analysis, calculated using the Kappa index was 0.81, indicating good agreement between observers. The quality of indicators for the selected studies was as follows: (i) the average methodological quality score was 82.55%. (ii) Two articles reached the maximum score of 100%; (iii) no study obtained a score below 50%. (iv) Six studies obtained a score between 50% and 75% (good methodological quality); and (v) 18 articles reached a rating of >75% (excellent methodological quality).

Regarding the items, criteria 1, 3, 4, 8, 9, 10, 11 and 14 obtained a coefficient of kappa = 1. Discrepancy between observers was obtained in criteria 2, 5, 6, 7, 12, 13, 15 and 16. Still values above 50% were found. Five items were mainly related with methodological deficiencies in the selected studies. (i) Criterion 4, where 20.41% of studies did not describe the sample in detail. (ii) Criterion 5, where 36.73% of studies sizes sample were not classified. (iii) Criterion 12, where 20.41% of articles did not inform about practice. (iv) Criterion 13, where 57.14% of cases did not report about dropouts. (v) Criterion 16, where 59.18% of articles did not clearly acknowledge the limitations of the study.

About Goal, the studies with the highest quality (85.42%) are those carried out on competition and training together. The category where the highest quality of studies was found was U18 (88.75%), finding lower levels in training categories. Depending on the level, the lowest quality levels were obtained in the state (81.81%) compared to national and international (87.5%). Regarding the type of load analysed in the investigations, those where the external load was exclusively analysed obtained higher quality values (88.75%); however, in articles where the internal load was analysed, mainly subjectively, the low quality (80%). Therefore, those studies that used objective instruments for load analyses were studies of higher quality (88.28%).

## 4. Discussion

The aim of this research, based on a systematic review, was to analyse the scientific production on women’s basketball. Studies where the load supported by the female players during practice and competition was investigated and evaluated. In this way, the intention was to identify the trends, samples, variables and instruments used, as well as the designs and research procedures used for this topic. The main results show that, in women’s basketball, competition has been mostly analysed, specifically by 48.15% of the studies evaluated. Only 14.81% of the studies have analysed training and competition together. This is a problem, since examining the demands imposed on the players, from both training and competition, provide a context to create an optimal training environment [16].

### 4.1. Competition Demands

Sports competition analysis is the most demanded. There are many hypotheses based on results, game statistics, quarters, playing position, etc. With regard to women’s basketball, the reviewed articles are responsible for characterizing sports competition in different levels (2, 3, 8, 13, 15, 17, 19, 21, 22, 23, 24). Competition in women’s basketball is defined to be able to replicate the demands during sports training. However, if the training is not analysed, it is impossible to know if the demands that generate are according to those in competition. The studies that have obtained a higher score regarding its quality have been those that jointly investigate training and competition, for which reason, it is considered of vital importance to increase the production of this type of research. Concerning demands, although the requirements of the competition in men’s basketball are greater than those of women’s basketball, Stojanović, Stojiljković, Scanlan, Dalbo, Berkelmans and Milanović [15] establish that the demands are similar, according to the level of competition in which the sample is located. The distance travelled during a match is similar; however, while women cover greater distances running, men cover greater distances dribbling. Female players performed at greater running work-rates than male players. This may be due to the fact that male basketball games have a longer duration (more possessions); therefore, a higher frequency of activities translates to a greater number of movement changes (22). Regarding the internal load variables, no significant differences have been established between sexes.

### 4.2. Training Demands

During training, load has been analysed according to the type of situations used in tasks, which in the reviewed articles are evaluated according to their characteristics and type of game situation employed (6, 7, 12, 14, 26). It is common to reduce the number of players in training tasks, as well as the size of the playing area or the track. These types of situations are known as small-sided games, and are arousing more interest in the scientific and sports communities [24]. Therefore, the types of training situations have been evaluated in order to compare them with demands generated by competition and in an attempt to try to match them. However, it has only been found that the demands of training equal or exceed those in matches or conditioning exercises (11, 12, 13, 16). Depending on the type of game situation used (small-sided games or full game), the load supported by the players is different. In women’s basketball, kinematic demands, as well as the more intense cardiac values, depend on the type of playing situation and competition is the most demanding condition (12, 16). The loads more similar to the competition are given in the 5 vs. 5 training tasks; however, higher values were always recorded in competition. The analysed studies, whose objectives have been the analyses of training, have obtained the lowest quality values compared to competition (this being a great limitation). The study of training is considered of vital importance as well as that of competition, since it is the means of improving the performance of athletes [25].

### 4.3. Category and Level

The most analysed category has been senior, finding that research in training categories (U15 and U16) is very limited (7.4% each). The vast majority of studies are carried out on state and national level samples (51.9% and 41.4%, respectively). In lower categories (U14–U18), analysis of training sessions is more common, while in senior categories (amateur and elite) the studies are more frequent on sports competition. It is important to highlight that, although the most carried out studies are at the state level, they are the lowest quality compared to national and international studies. A review of the literature found significant differences in relation to the level and category of the sample. In competition, the distance covered by the athletes is similar (5–6 km per game) but there are differences between categories related to the intensity of play. Higher-level players spend more time in more intense work areas and vary between them more intermittently (21). The same happens with the physiological responses to the different types of activity, when the activity is more intense the internal load increases. In turn, it is found that in U14 and U16 there are fewer studies and those that exist are less complete.

### 4.4. Load Demands and Evaluations

The load has been analysed in female basketball during training and sports competitions. The most common is the analysis of the internal and external load together, even so, during the competition, the analysis of the external load has been more investigated (30.77%). Furthermore, studies that tried to provide information on external load have obtained a higher quality than the rest. Even so, it is considered interesting to increase the quality of the studies carried out with external and internal load together, since they provide a more global vision of the demands of the game in athletes [8,15].

Specifically, for the analysis of internal load during training in women’s basketball, the heart rate (1, 7, 12, 13, 14, 18) and subjective variables, such as the RPE (Rating of Perceived Exertion) scale or questionnaires (4, 7, 10, 14, 20, 25), have been mainly analysed. With respect to analysis of the internal load in women’s basketball, the authors have established in competition a HRavg of 162–173 ppm, a HRmax of 188–195 ppm for % HRMax 82.4%–92.5% (1, 7, 13, 12, 18). As for lactate measurements, values have been obtained between 3.7–5.2 mmoles (8, 9, 21).

Inertial devices and TMA are the most commonly used tools for external load analysis. Information is extracted about the activity that is being carried out on frequency of actions, and their duration or intensity. In addition, data are obtained relating to distances travelled and changes in movement. In women’s basketball, Scanlan et al. (21) record that the players cover an average of 5214 ± 315 m per game. The intensity at which they work during the race is 30.2%–39.3% walking, 12.8%–24% jogging, 4.9%–11% running, and 0.6%–7.8% sprinting (3, 5, 21, 22). An average of 576–652 changes of movements are made, producing one every 2.49–2.82 s (3, 5, 8); however, Scalan et al. (21) collected 1752 movement changes per game in amateur Australian players. Regarding high-intensity actions between 19–71 jumps per game (1–1.77 jumps per minute) and between 44 and 49 sprints per game (3, 5, 12) have been reported, although, Scalan et al. (21) recorded an average of 108 sprints per game. These previous studies that have examined the load using time motion video analysis led to controversial conclusions. For this reason, in the most current studies, measurements derived from accelerometers, GPS and other devices are used as alternative, objective and reliable methods to evaluate the external training load.

The evaluation of loads analysed by these devices have only been carried out in studies on training sessions due to the negative posture of federations as to their use during the sports competition. Load has been evaluated in sports training with the main objective of comparing the game situations used in the training tasks. Significant differences have been found between small-sided games (2 vs. 2, 3 vs. 3) compared to the full game (4 vs. 4, 5 vs. 5), the former causing more demands in full court tasks (6, 7). These authors have established differences in the distance travelled, the average speed, PlayerLoad (PL), the speed ranges in accelerations, heart rate, sprints and jumps. Herran et al. (6) recorded 5-min games in situations of 3 vs. 3 and 5 vs. 5, obtaining differences in the total distance values (249.6 vs. 209.35 m respectively), average speed (49.9 vs. 41.8 m/min) and PlayerLoad (47.6 vs. 41.8). On the other hand, Kluseman et al., (7) studied tasks lasting 10 min in situations of 2 vs. 2 and 4 vs. 4, obtaining values of % RH (86.4% vs. 83.5%), number of jumps (26.1 vs. 16.6), in addition to observing activity of greater intensity in 2 vs. 2 situations. Reina et al., [12] did not find significant differences between the training situations; however, they also evaluated the physical requirements in competition where they found higher levels of intensity regarding internal load (HRmax and HRavg) and distance travelled.

## 5. Limitations

Some limitations should be addressed when considering this research on the training load and competition demands in women’s basketball. First, it should be noted that this review is not intended to accurately provide the demands performed during competition and training in women’s basketball. This review aims to establish where there are declines to encourage researchers to create scientific evidence in this poorly studied population. When differentiating between levels, it is found that the number of studies in training categories is lower, making it difficult to define them adequately in order to create specific training patterns. The same applies to the type of load analysed and the instrument used. Depending on the level and purchasing power, in some studies, the training load will be more precisely quantified. Being important, a great investment in training categories and women’s basketball. Finally, regarding the qualitative analysis, it was found that, mainly, the studies in training categories (U15 and U16) together with the studies carried out in level state were of a lower quality. This is an aspect to take into account since, on the one hand, it is important to individualize training according to age and, on the other hand, the largest number of existing studies is in level state.

## 6. Conclusions

Currently, conditions for obtaining relevant scientific information that allows us to monitor and establish workloads in basketball players are optimal, and this systematic review establishes a profile of knowledge about the load supported by women’s basketball players. It has been possible to verify that the bibliography dedicated to this topic is scarce, being nourished from data coming from men’s basketball, but at the same time, proposals for the study of women’s basketball are being established.

Studies are necessary that propose individualized training for this type of population, that not only characterize the hormonal profile of the women athletes or evaluate the etymology of their injuries, but also prescribe specific training to prevent injuries and, consequently, to increase their performance. It is important to highlight the use of inertial devices today. This type of device is booming, with tendency to be the most used instrument by researchers for the analysis of sports performances in the future. For a better description of women’s basketball, there is a need to jointly investigate sports training and competition, to study the training categories and to use micro technology that guarantees obtaining objective and reliable data. Finally, this review takes into consideration the evaluation of quality of the studies, a risk-of-bias form developed to the specific context.

## Figures and Tables

**Table 1:** Level and Type of Load by Goal.

Variables			Level			Total	Type of Load			Total
			State	National	International		External	Internal	Both	
Goal	T	*n*	6	4	0	10	2	2	6	10
		% Goal	60.00	40.00	0.00	100.00	20.00	20.00	60.00	100.00
		% L/TL	42.86	33.33	0.00	37.04	28.57	40.00	40.00	37.04
	C	*n*	5	7	1	13	4	2	7	13
		% Goal	38.46	53.85	7.69	100.00	30.77	15.38	53.85	100.00
		% L/TL	35.71	58.33	100.00	48.15	57.14	40.00	46.67	48.15
	B	*n*	3	1	0	4	1	1	2	4
		% Goal	75.00	25.00	0.00	100.00	25.00	25.00	50.00	100.00
		% L/TL	21.43	8.33	0.00	14.81	14.29	20.00	13.33	14.81
Total		*n*	14	12	1	27	7	5	15	27
		%	51.85	44.44	3.70	100.00	25.93	18.52	55.56	100

Abbreviations: T (training); C (competition); B (both).

**Table 2:** Instruments by Goal.

Variables			Instruments								Total
			HRB	GPS	ID	TMA	BM	SS	TEST	MIN	
Goal	T	*n*	4	1	3	1	0	5	4	2	10
		% Goal	40.00	10.00	30.00	10.00	0.00	50.00	40.00	20.00	
		% I	30.77	50.00	30.00	16.67	0.00	71.43	66.67	66.67	
	C	*n*	7	1	4	5	3	1	2	1	13
		% Goal	53.85	7.69	30.77	38.46	23.08	7.69	15.38	7.69	
		% I	53.85	50.00	40.00	83.33	100.00	14.29	33.33	33.33	
	B	*n*	2	0	3	0	0	1	0	0	4
		% Goal	50.00	0.00	75.00	0.00	0.00	25.00	0.00	0.00	
		% I	15.38	0.00	30.00	0.00	0.00	14.29	0.00	0.00	
Total		*n*	13	2	10	6	3	7	6	3	27
		%	48.15	7.41	37.04	22.22	11.11	25.93	22.22	11.11	100

Abbreviations: T (training); C (competition); B (both); I (instruments); HRB (heart rate band); GPS (global positioning system); ID (inertial devices); TMA (time motion analysis); BM (blood markers); SS (subjective scale); TEST (fatigue tests); MIN (minutes).

**Table 3:** Variables by Goal.

Variables			Variables									Total
			HR	SP	J	MOV	SL	D	PL	ACC	LAC	
Goal	T	*n*	5	2	0	2	4	0	2	1	0	10
		% Goal	50.00	20.00	0.00	20.00	40.00	0.00	20.00	10.00	0.00	
		% V	38.46	28.57	0.00	18.18	50.00	0.00	33.33	20.00	0.00	
	C	*n*	6	5	5	7	3	5	2	4	1	13
		% Goal	46.15	38.46	38.46	53.85	23.08	38.46	15.38	30.77	7.69	
		% V	46.15	71.43	71.43	63.64	37.50	71.43	33.33	80.00	100	
	B	*n*	2	0	2	2	1	2	2	0	0	4
		% Goal	50.00	0.00	50.00	50.00	25.00	50.00	50.00	0.00	0.00	
		% V	15.38	0.00	28.57	18.18	12.50	28.57	33.33	0.00	0.00	
Total		*n*	13	7	7	11	8	7	6	5	1	27
		%	48.15	25.93	25.93	40.74	29.63	25.93	22.22	18.52	3.70	100

Abbreviations: T (training); C (competition); B (both); V (variables); HR (heart rate); SP (speed profile); J (jump), SL (subjective load); D (distance); PL (player load); ACC (accelerations); LAC (blood lactate).

## Supplementary Materials

The following are available online at https://www.mdpi.com/1660-4601/17/8/2639/s1, Table S1: Summary of articles.
