# Supplementary material for: Training and Competition Load in Female Basketball: A Systematic Review
Source: Int J Environ Res Public Health. 2020 Apr 12;17(8):2639. doi: 10.3390/ijerph17082639 (PMC7215482; doi:10.3390/ijerph17082639)
Supplement: Supplementary file 1 [file ijerph-17-02639-s001.pdf]

*Supplementary Material*

**ATTACHED FILE I. List of items**


1. Atl, H., Köklü, Y., Alemdaroglu, U., & Koçak, F. Ü. (2013). A comparison of heart rate response and frequencies of technical actions between half-court and full-court 3-a-side games in high school female basketball players. *The Journal of Strength Conditioning Research 27*(2), 352-356.

2. Batalla, A., Bofill, A. M., Montoliu, R., & Corbi, F. (2018). Relationship between Heart Rate and the Scoreboard during a Relegation Playoff. *Apunts. Educació Física i Esports* (132), 110-122.

3. Conte, D., Favero, T. G., Lupo, C., Francioni, F. M., Capranica, L., & Tessitore, A. (2015). Time-motion analysis of Italian elite women's basketball games: individual and team analyses. *The Journal of Strength Conditioning Research 29*(1), 144-150.

4. Cruz, I., Pereira, L. A., Kobal, R., Kitamura, K., Cedra, C., Loturco, I., & Abad, C. C. C. (2018). Perceived training load and jumping responses following nine weeks of a competitive period in young female basketball players. *PeerJ, 6*, e5225.

5. Delextrat, A., Badiella, A., Saavedra, V., Matthew, D., Schelling, X., & Torres-Ronda, L. (2015). Match activity demands of elite Spanish female basketball players by playing position. *J International Journal of Performance Analysis in Sport, 15*(2), 687-703.

6. Herrán, A., Usabiaga, O., & Castellano, J. (2017). Comparación del perfil físico entre 3x3 y 5x5 en baloncesto formativo. *Revista Internacional de Medicina y Ciencias de la Actividad Física y el Deporte*

7. Klusemann, M. J., Pyne, D. B., Foster, C., & Drinkwater, E. J. (2012). Optimising technical skills and physical loading in small-sided basketball games. *Journal of Sports Sciences 30*(14), 1463-1471.

8. Matthew, D., & Delextrat, A. (2009). Heart rate, blood lactate concentration, and time–motion analysis of female basketball players during competition. *Journal of Sports Sciences, 27*(8), 813-821.

9. Montgomery, P. G., & Maloney, B. D. (2018). 3× 3 Basketball: Performance Characteristics and Changes During Elite Tournament Competition. *International Journal of Sports Physiology Performance 13*(10), 1349-1356.

10. Paulauskas, H., Kreivyte, R., Scanlan, A. T., Moreira, A., Siupsinskas, L., Conte, D. J. I. j. o. s. p., & performance. (2019). Monitoring workload in elite female basketball players during the in-season phase: weekly fluctuations and effect of playing time. *International Journal of Sports Physiology Performance 14*(7), 941-948.

11. Peterson, K. D., & Quiggle, G. T. (2017). Tensiomyographical responses to accelerometer loads in female collegiate basketball players. *Journal of Sports Sciences, 35*(23), 2334-2341.

12. Reina, M., Mancha, D., & Ibáñez, S. J. (2017). ¿ Se entrena como se compite? Análisis de la carga en baloncesto femenino. *Revista de Psicología del Deporte 26*(1), 9-13.

13. Reina, M., García-Rubio, J., Feu, S., & Ibáñez, S. J. (2018). Training and Competition Load Monitoring and Analysis of Women's Amateur Basketball by Playing Position: Approach Study. *Frontiers in psychology, 9*.

14. Reina, M., Mancha-Triguero, D., García-Santos, D., García-Rubio, J., & Ibáñez, S. J. (2019). Comparación de tres métodos de cuantificación de la carga de entrenamiento en baloncesto.[Comparison of three methods of quantifying the training load in basketball]. *Revista internacional de ciencias del deporte, 15*(58), 368-382.

15. Reina, M., García-Rubio, J., Pino-Ortega, J., & Ibáñez, S. J. (2019). The Acceleration and Deceleration Profiles of U-18 Women's Basketball Players during Competitive Matches. *Sports, 7*(7), 165.

16. Reina, M., García-Rubio, J., Antúnez, A., Courel-Ibáñez, J. & Ibáñez, S.J (2019). Load Variability of Training Sessions and Competition in Female Basketball. *Journal of Sport Psychology*, *28* (1), 93–99.

17. Reina, M., Rubio, J. G., Antúnez, A., & Ibáñez, S. J. (2020). Comparación de la carga interna y externa en competición oficial de 3 vs. 3 y 5 vs. 5 en baloncesto femenino (Comparison of internal and external load in official 3 vs. 3 and 5 vs. 5 female basketball competitions). *Retos. Nuevas Tendencias En Educación Física, Deporte Y Recreación, 37*(37), 400-405.

18. Sánchez-Sánchez, J., Carretero, M., Valiente, J., Gonzalo-Skok, O., Sampaio, J., & Casamichana, D. (2017). Heart rate response and technical demands of different small-sided game formats in young female basketballers.[Respuesta de la frecuencia cardíaca y demanda técnica en diferentes formatos de juegos reducidos realizados por jugadoras jóvenes de baloncesto]. *Revista internacional de ciencias del deporte, 14*(51), 55-70.

19. Sanders, G. J., Boos, B., Rhodes, J., Kollock, R. O., & Peacock, C. A. (2018). Competition-Based Heart Rate, Training Load, and Time Played Above 85% Peak Heart Rate in NCAA Division I Women's Basketball. *Journal of Strength Conditioning Research*

20. Sansone, P., Tschan, H., Foster, C., & Tessitore, A. (2018). Monitoring Training Load and Perceived Recovery in Female Basketball: Implications for Training Design. *Journal of Strength Conditioning Research*.

21. Scanlan, A. T., Dascombe, B. J., Kidcaff, A. P., Peucker, J. L., & Dalbo, V. J. (2015). Gender-specific activity demands experienced during semiprofessional basketball game play. *International Journal of Sports Physiology Performance 10*(5), 618-625.

22. Scanlan, A. T., Dascombe, B. J., Reaburn, P., & Dalbo, V. J. (2012). The physiological and activity demands experienced by Australian female basketball players during competition. *Journal of Science Medicine in Sport 15*(4), 341-347.

23. Staunton, C., Wundersitz, D., Gordon, B., Custovic, E., Stanger, J., & Kingsley, M. (2018). The effect of match schedule on accelerometry-derived exercise dose during training sessions throughout a competitive basketball season. *Sports, 6*(3), 69.

24. Staunton, C., Wundersitz, D., Gordon, B., & Kingsley, M. (2018). Accelerometry-derived relative exercise intensities in elite women's basketball. *International Journal of Sports Medicine 39*(11), 822-827.

25. Vallés-Ortega, C., Fernández-Ozcorta, E., & Fierro-Suero, S. (2017). Patrón fatiga-recuperación en una competición de alta densidad competitiva en Baloncesto Femenino Junior. *Cuadernos de Psicología del Deporte, 17*(3), 183-188.

26. Vallés-Ortega, C., Fernández-Ozcorta, E., & Suero, S. F. (2017). Relación entre la complejidad técnico-táctica en la sesión de entrenamiento y la carga interna en baloncesto femenino. *SPORT TK*, 163-168.

| **Table S1.** Summary of articles | | | | | | | | | |
|---|---|---|---|---|---|---|---|---|---|
| **STUDY** | | **PARTICIPANTS** | **YEAR** | **LEVEL** | **GOAL** | **SAMPLE** | **LOAD** | **INSTRUMENTS** | **VARIABLES** |
| **1** | **Atl et al. (2013)** | 12 female basketball players | U16 (15.56±05 years) | State | Training | half-court and full-court 3-a-Side training games | Internal | Heart rate band (Polar Team Sport System) | % Heart rate peak |
| **2** | **Batalla et al. (2018)** | 10 female basketball players | Senior (21.3±2.71 years) | State | Competition | 10 official games | Internal | Heart rate band (Suunto Team Pack®) | % Heart rate peak |
| **3** | **Conte et al. (2015)** | 12 female basketball players | Senior (27±6.4 years) | National | Competition | 5 official games | External | Video recordings (Sony HD AVCHD HDR CX115) | Activity categories (Standing/walking, jogging, running, sprinting, jumping, low, moderate and high-intensity-specific movements) |
| **4** | **Cruz et al. (2018)** | 10 young female basketball players | U18 (17.2±0,4) | State | Training | 9-weeks training | Internal and external | RPE<br>TQR<br>CMJ | |
| **5** | **Delextrat et al. (2015)** | 42 female basketball players belonging to six teams | Senior (25.9±4.3) | National | Competition | 3 official games from each team | External | Video recordings (LINCE) | Activity categories intensity, specific movements and static exertion. |
| **6** | **Herran et al. (2017)** | 10 female basketball players | U16 (15 ±1.0 years) | State | Training | 3x3 and 5x5 training games | External | GPS ((MinimaxX v.4.0, Catapult) | Distance, average speed, Player Load and ranges of acceleration |

| | | | | | | | | | |
|---|---|---|---|---|---|---|---|---|---|
| 7 | **Klusemann et al. (2012)** | 16 female basketball players | U18 (17.4+0.7 years) | National | Training | 19 training games | Internal | Heart rate band (Suunto™) RPE Video recordings (SportsCodeElite) | %Heart rate peak, percentage of time spent in Zone 4 and Zone 5. Tecnical events. Subjective load |
| 8 | **Matthew et al. (2009)** | 9 female basketball players | Senior (25.8±2.5 years) | National | Competition | 9 official games | Internal and external | Heart rate band (Polar S810) Video recordings (VC-x400) Blood samples (Analox LM5) | Time motion analysis (jumps, sprints, activity demands, intensity), heart rate average and 85% of heart rate peak, blood lactate |
| 9 | **Montgomery et al. (2018)** | 201 female basketball players in 3x3 competition | Senior (22.9 ± 5.6 years) | International | Competition | 3x3toutnament | Internal and external | GPS and heart rate band (OptimEye S5, Catapult) Blood samples (Lactate Scout™), RPE | CoD, accelerations and decelerations, jumps, PlayerLoad, Blood Lactate, Heart rate peak and average. |
| 10 | **Paulauskas et al. (2019)** | 29 female basketball players belonging to seven teams | Senior (21 ± 5 years) | National | Training | 24-weeks training | Internal and external | RPE and Duration (minutes). | Total weekly TL, weekly TL, weekly GL, chronic workload, training monotony |
| 11 | **Peterson et al. (2017)** | 5 female basketball players | Senior (20 ± 1.0 years) | National | Training | 20-weeks training | External | Inertial devices (Catapult Optimeye S5) and TMG | Player Load™, IMA™, Tc, and Dm. |

| | | | | | | | | | |
|---|---|---|---|---|---|---|---|---|---|
| | | | | | | | | assessments (TMG-BMC d. o.o., Ljubljana, Slovenia) | |
| 12 | **Reina et al. (2017)** | 10 female basketball players | Senior (21.7±3.65 years) | State | Training and competition | 8 official games and 22 training sessions | Internal and external | Heart rate band (Garmin™) and inertial device (Wimu™) | Heart rate peak and average. Impacts, Steps and Jumps per minute |
| 13 | **Reina et al. (2018)** | 10 female basketball players | Senior (21.7±3.65 years) | State | Training and competition | 8 official games and 47 5 vs.5 training tasks | Internal and external | Heart rate band (Garmin™) and inertial device (Wimu™) | Heart rate peak and average, % Heart rate peak and work zones. Number of Impacts, Steps, Jumps and Player Load |
| 14 | **Reina et al. (2019)a** | 10 female basketball players | Senior (21.7±3.65 years) | State | Training | 22 training sessions (120 tasks) | Internal and external | Heart rate band (Garmin™) and inertial device (Wimu™) SIATE | % Heart rate peak, PlayerLoad, subjective load |
| 15 | **Reia et al. (2019)b** | 48 female players belonging to four teams | U18 | State | Competition | 12 official games | External | Inertial device and Ultra Wided Band (Wimu™) | Number of accelerations and decelerations. Duration (ms), Start speed (km/h) and Acceleration peak (m/s2) of each one. Intensity ranges (m/s2). |

| | | | | | | | | | |
|---|---|---|---|---|---|---|---|---|---|
| 16 | **Reina et al. (2019)c** | 12 female basketball players | U15 | State | Training and competition | 8 official games and 35 training sessions | External | Inertial device (Wimu™) | Player Load |
| 17 | **Reina et al. (2020)** | 10/ 8 female basketball players | Senior / U18 | State/National | Competition | 8 official games (5x5) / 6 official games (3x3) | Internal and external | Heart rate band (Garmin™) and inertial device (Wimu™) | Heart rate peak and average, % Heart rate peak and work zones. Number of steps, jumps and impacts per minute |
| 18 | **Sanchez-Sanchez et al. (2017)** | 6 female basketball players | U15 (14.3 ± 0.5 years) | State | Training | 2 training sessions | Internal | Heart rate band (Polar Team System 2) YOYO test | Heart rate peak and average, % Heart rate peak |
| 19 | **Sanders et al. (2018)** | 10 female basketball players | Senior (19.8±1.3 years) | National | Competition | 31 official games | Internal | Heart rate band (Polar Global) | Heart rate peak and average, % Heart rate peak and work zones. |
| 20 | **Sansone et al. (2018)** | 13 female players | Senior (22.6±3 years) | National | Training and competition | 40 training sessions, 2 friendly games and 12 official games . | Internal | RPE and TQR scales | |
| 21 | **Scanlan et al. (2015)** | 12 female basketball players | Senior (21.2 ± 3.1) | National | Competition | 3 official games | External | Video recordings (Basler A602FC) | Activity frequencies, durations, and distances |

| | | | | | | | | | |
|---|---|---|---|---|---|---|---|---|---|
| 22 | **Scanlan et al. (2012)** | 12 female basketball players | Senior (22.0±3.7 years) | State | Competition | 8 official matches | Internal and external | Heart rate band (Polar Electro) Video recordings (Basler A602FC) Blood samples (Accusport Lactate Analyser) | Activity frequencies, total durations and total distances, heart rate average, % heart rate peak, blood lactate. |
| 23 | **Staunton et al. (2018)a** | 10 female basketball players | Senior (27 ± 5 years) | National | Competition | 18 official games | Internal and external | Tri-axial accelerometer (Link). Respiratory parameters (Oxycon Mobile) | Peak VO2; duration (%) spent in each intensity zone; Frequency, average bout duration and maximum bout. |
| 24 | **Staunton et al. (2018)b** | 9 female basketball players | Senior (27 ± 5 years) | National | Training | 6-weeks training with 3 sessions per week | Internal and external | Accelerometer (ActiLife) VO2 (LabVIEW) | Total duration (%) spent in each intensity zone. Exercise dose (Impulse) for easy, medium, and hard match schedules |
| 25 | **Vallés et al. (2017)a** | 12 female basketvall players | U18 (17.08±0.67) | State | Competition | 6 official games | Internal and external | Borg TQR Wellness Minutes CMJ | |

| | | | | | | | | | |
|---|---|---|---|---|---|---|---|---|---|
| **26** | **Vallés et al. (2017)b** | 12 female basketvall players | U18 (17.08±0.67) | State | Training | Training sessions throught championship | Internal and external | Borg<br>TQR<br>Wellness<br>Minutes<br>CMJ | |
